# Valuing Mortality Risk Reductions in Global Benefit-Cost Analysis

**DOI:** 10.1017/bca.2018.26

**Published:** 2019-01-15

**Authors:** Lisa A. Robinson, James K. Hammitt, Lucy O’Keeffe

**Affiliations:** 1 Harvard T.H. Chan School of Public Health, Center for Health Decision Science and Center for Risk Analysis, 718 Huntington Avenue, Boston, Massachusetts 02115, USA; 2 Toulouse School of Economics, Université Toulouse Capitole, 21, allée de Brienne, 31000 Toulouse, France

**Keywords:** low- and middle-income countries, value of mortality risk reductions, value per statistical life, willingness to pay

## Abstract

The estimates used to value mortality risk reductions are a major determinant of the benefits of many public health and environmental policies. These estimates (typically expressed as the value per statistical life, VSL) describe the willingness of those affected by a policy to exchange their own income for the risk reductions they experience. While these values are relatively well studied in high-income countries, less is known about the values held by lower-income populations. We identify 26 studies conducted in the 172 countries considered low- or middle-income in any of the past 20 years; several have significant limitations. Thus there are few or no direct estimates of VSL for most such countries. Instead, analysts typically extrapolate values from wealthier countries, adjusting only for income differences. This extrapolation requires selecting a base value and an income elasticity that summarizes the rate at which VSL changes with income. Because any such approach depends on assumptions of uncertain validity, we recommend that analysts conduct a standardized sensitivity analysis to assess the extent to which their conclusions change depending on these estimates. In the longer term, more research on the value of mortality risk reductions in low- and middle-income countries is essential.

**JEL classifications:** D6; I1; H4; Q5.

## 1 Introduction

Increasing life expectancy is a major goal of many policies. As a result, the value of mortality risk reductions has been extensively studied and several organizations and individuals have developed recommendations for estimating these values in benefit-cost analysis. However, both the recommendations and the underlying research primarily address high-income settings, raising questions about the extent to which the results are applicable to low- and middle-income countries. The recommendations are also diverse, reflecting differing methodological choices as well as differing policy contexts.

In this paper, we develop recommendations for valuing mortality risk reductions in benefit-cost analysis of policies to be implemented in low- and middle-income countries, combining previous work with additional review of individual studies. We introduce related concepts and methods, describe approaches recommended in recent analyses, and discuss the results of our review. We focus on developing population-average estimates that can be applied to policies with nationwide impacts, noting how these estimates can be adapted for policies that address different age groups within that population. Similar approaches can be used to adjust the estimates for subgroups with income levels that differ from the national average.

As conventionally conducted, benefit-cost analysis is based on respect for individual preferences. Value is measured by the amount of money individuals affected by a policy are willing to exchange for the benefits they receive. Money is not important *per se*; rather, it reflects the resources available to spend on risk reductions and other goods and services. Spending on mortality risk reductions means that individuals – and the society of which they are a part – will have fewer resources available to spend on other things. Understanding these preferences is useful regardless of whether the policy decision is based on a comparison of benefits and costs or on other grounds.

The concept of individual willingness to pay (WTP) for changes in one’s own risk has been obscured by the language economists use to describe these values. A reduction in mortality risk that accrues throughout a population decreases the expected number of deaths within a time period. Hence the total monetary value of the individual risk reductions can be summarized as the value per expected life saved, generally described as the value per “statistical” life (VSL). VSL is often misinterpreted. It is not the value that the individual, the society, or the government places on averting a death with certainty. Rather, it represents the rate at which an individual views a change in money available for spending as equivalent to a small change in his or her own mortality risk.

Over the years, many alternative terms have been suggested, but none have been widely accepted or used.^1^ Some researchers have recommended terms such as the “value per standardized mortality unit” (VSMU) (Jamison et al., 2013) or the “value of reduced mortality risk” (VRMR) (Dockins et al., 2018).^2^ Because these terms drop the reference to “statistical life,” the size of the risk reduction to which the value applies must also be defined. For example, Jamison et al. (2013) define the VSMU as equivalent to individual WTP for a mortality risk reduction of 1 in 10,000. Others (e.g., Howard, 1989) have used the term “micro-mort” to refer to the value of a 1 in 1 million risk change. To connect the concepts and estimates presented in this paper with the well-established VSL literature, we use the term VSL where relevant, but more generally refer to the value of mortality risk reductions.

A closely-related concept is the value per statistical life year (VSLY): the rate at which an individual is willing to trade small changes in his or her own life expectancy for spending on other goods and services. While the VSLY can be estimated empirically, little research is available. Instead, a constant VSLY is typically derived from a VSL estimate and used to adjust the analytic results to reflect the effects of differences in age or life expectancy.

## 2 Valuation concepts and methods

The starting point for valuing mortality risk reductions is typically an estimate of the change in the likelihood of death in a defined time period for individuals affected by a policy. This risk change can be aggregated over the affected population to calculate the expected change in the number of deaths in that period (that is, the deaths delayed to later periods). In most cases, the identity of those whose deaths would be postponed is not known either before or after the policy is implemented.

Consistent with the benefit-cost analysis framework, the value of these risk reductions is based on individuals’ willingness to trade spending on other goods and services for reductions in their own risks. VSL is the ratio of the amount of money an individual would give up in exchange for a small reduction in mortality risk (in a specified period), such that he or she is no better and no worse off with the reductions in mortality risk and the money remaining than without the exchange. In other words, VSL is an individual’s marginal rate of substitution between income (or more accurately, wealth) and the risk of dying in a defined time period. For small changes in risk, VSL can be approximated by dividing an individual’s WTP for a specified risk change by that risk change.^3^

An individual’s WTP presumably encompasses all of the impacts of the risk change on his or her well-being – including both pecuniary effects (such as avoided out-of-pocket medical costs and losses in future earnings as well as increased future opportunities for consumption) and non-pecuniary effects (such as continuing to experience the joys of life itself and delaying the pain and suffering associated with dying). It also reflects the trade-off between spending while alive and bequeathing money to others at death. These values vary across individuals and across different types of risk; there is no single value that is applicable to all contexts.

Because mortality risk reductions are not directly bought and sold in the marketplace, WTP estimates are usually derived using stated- or revealed-preference methods. Stated-preference studies typically employ survey techniques to ask respondents about their WTP for an outcome under a hypothetical scenario. An advantage of these methods is that the scenario can be tailored to reflect the characteristics of the populations and risks addressed by a specific policy. A disadvantage is that the survey must be carefully designed to ensure that respondents understand the scenario and provide valid responses. Revealed-preference methods instead infer the value of nonmarket outcomes from observed behaviors and prices for related market goods. For example, wage-risk studies (often referred to as hedonic-wage studies) examine the change in compensation associated with jobs that involve differing risks of fatal injury. These studies use statistical methods to separate the effects of mortality risk on wages from the effects of other job and personal characteristics. While revealed-preference methods have the advantage of reflecting actual behavior, it can be difficult to find a market good for which adequate, high-quality data are available that allow analysts to separate the value of the risk reduction from the effects of other factors that influence its price.

Conducting new primary research requires substantial time and expense; typically analysts instead rely on existing valuation studies. This approach is referred to as “benefit transfer” (or sometimes “value transfer”) to indicate that the populations and policies studied are not necessarily identical to the population and policy considered in a particular benefit-cost analysis. Similar to the process used to estimate other parameter values, such transfers involve carefully reviewing the literature to identify high-quality studies that are suitable for use in a particular context, and determining whether and how to combine and adjust the results prior to application.

Because any individual study will have both advantages and limitations, analysts often prefer to draw on multiple studies when estimating values. The synthesis approaches used most frequently in the VSL literature include criteria-driven systematic reviews and meta-analyses; expert elicitation and structural models may be used but are applied infrequently (see Robinson & Hammitt, 2015). A systematic review follows a well-defined, structured approach to identify studies and to evaluate their quality and applicability to the policy of concern. This review may be used to directly identify a range of estimates for application, or may provide a starting point for additional quantitative assessment, such as through the use of meta-analysis.

Meta-analysis applies statistical methods to combine the results of multiple studies and investigate sources of variation. The results can be used to develop reduced form equations that allow analysts to transfer values across contexts based on relatively few input variables. While meta-analysis can control for differences across studies statistically, it is useful to begin with a systematic review to select studies of sufficient quality that address reasonably similar outcomes. A major problem in applying these and other research synthesis approaches is the lack of consistent reporting standards in the VSL literature; often data on the key variables of interest are not provided in the articles documenting the study results.

## 3 Current practices

The value of mortality risk reductions is relatively well studied; recent reviews suggest that over 200 studies have been completed globally. Because of the importance of these estimates, substantial attention has been paid to developing criteria for evaluating study quality and applicability, particularly in high-income settings. However, relatively few studies have been conducted in low- and middle-income countries.

When evaluating policies to be implemented in low- and middle-income countries, benefit-cost analysts typically rely on one of two approaches: (1) they use the results of studies conducted in the country of concern if available; (2) they extrapolate from values from other countries (almost always of higher income), adjusting for income differences. While the first option is preferable when studies from the country are of sufficient quality, the paucity of research in many settings means that analysts often follow the second option. We first discuss the base values used in these extrapolations, then discuss the adjustments used for income differences.

### 3.1 Base values

The starting point for estimating values for low- and middle-income countries is often either values developed for use in national U.S. regulatory analyses or for application by OECD member countries. These estimates were derived at different times using differing approaches, but are each based on substantial review and evaluation of a large number of studies and are well established and widely used. In recent work, Viscusi and Masterman have suggested instead relying on a meta-analysis of U.S. wage-risk studies, which results in estimates within the same range as those applied by the U.S. regulatory agencies.

U.S. regulatory agencies typically develop their recommendations by reviewing the literature and identifying a range of values and a central estimate from selected studies (U.S. Environmental Protection Agency, 2010*a*; U.S. Department of Health and Human Services, 2016; U.S. Department of Transportation, 2016). Each agency uses different criteria and includes different studies in developing their estimates, but all rely primarily on wage-risk studies. The OECD has taken a different approach, focusing on stated-preference studies conducted globally and using meta-analysis to combine the results (OECD, 2012).

Comparing the monetary values requires translating them into the same currency for the same year. Rather than making this conversion, we compare the ratio of the reported values to gross national income (GNI) per capita for the country, in the same year as the VSL estimate.^4^ Although many different income measures could be used (see Hammitt, 2017), we rely on GNI per capita because consistently-derived estimates are available and easily accessible for a large number of countries, and because it is a broader measure than gross domestic product (GDP) per capita.

To translate values across currencies, we use purchasing power parity. Purchasing power parity is an index designed to represent what money can purchase in different economies. Compared with market-exchange rates, it better reflects the resources available to the population of each country when considering how these resources may be allocated across achieving mortality risk reductions and consuming other goods and services. One international dollar would buy a comparable quantity of goods and services in the country of concern as would a U.S. dollar spent in the United States. This means that U.S. GNI per capita is the same regardless of whether it is expressed in U.S. or international dollars.

Table 1 provides the estimates used by U.S. agencies and the OECD, expressed both as VSL and as WTP for a small risk change and compared to GNI per capita. The relationships between the U.S. values and income are similar across the three agencies; the relationship of the OECD estimate to income differs significantly.

**Table 1:** Comparison of VSL to GNI per capita: U.S. and OECD.

Source (dollar year)	VSL estimate (range)	GNI per capita	Ratio of VSL to GNI per capita	WTP for 1 in 10,000 risk change	WTP as percent of GNI per capita
**USEPA** (2010*a*) (2006 USD)	$7.4 million (± $4.7 million)	$47,390	156 (57, 255)	$740 ($270, $1210)	1.6% (0.6%, 2.6%)
**USDHHS** (2016) (2014 USD)	$9.3 million ($4.4 million, $14.2 million)	$56,160	166 (78, 253)	$930 ($440, $1420)	1.7% (0.8%, 2.5%)
**USDOT** (2016) (2015 USD)	$9.6 million ($5.4 million, $13.4 million)	$57,900	166 (93, 231)	$960 ($540, $1340)	1.7% (0.9%, 2.3%)
**OECD** (2012) (2005 USD)	$3.0 million ($1.5 million, $4.5 million)	$30,601	98 (49, 147)	$300 ($150, $450)	1.0% (0.5%, 1.5%)

USEPA = U.S. Environmental Protection Agency USDHHS = U.S. Department of Health and Human Services USDOT = U.S. Department of Transportation Note: The U.S. estimates are designed to address nationwide policies; the OECD estimate is intended for application to analyses that address all OECD countries. GNI per capita is reported for the same year as the estimate.

These recommended values change periodically as new studies are completed and researchers develop new insights into best practices. For example, the U.S. Environmental Protection Agency (USEPA) has proposed to update its estimates based on meta-analysis of selected studies and advice from its expert panels (U.S. Environmental Protection Agency, 2016; Khanna et al., 2017). In addition, researchers are now updating the database and meta-analysis that underlies the OECD estimates.^5^

Recently, Viscusi and Masterman recommended instead relying on meta-analysis of wage-risk studies that use data from the U.S. Census of Fatal Occupation Injuries (CFOI) (Viscusi, 2015*b*; Viscusi & Masterman, 2017*a*,*b*; Masterman & Viscusi, 2018; Viscusi, 2018).^6^ In this research, Viscusi and Masterman assess the effects of publication-selection bias, which occurs when researchers or journals reject estimates that fall outside of a range deemed acceptable. They find substantial evidence of such bias both in U.S. studies that rely on other sources of risk data and in datasets that include international sets of revealed- or stated-preference studies. Their recommended base estimate, derived from meta-analysis of U.S. studies that rely on CFOI data, is a VSL of $9.6 million in 2015 U.S. dollars.

This U.S. estimate is very similar to the values currently used by U.S. regulatory agencies. If we update the values in Table 1 to 2015 dollars, using the approaches for adjusting for inflation and real income growth followed by each organization, the U.S. central estimates are all between $9 million and $10 million. The OECD estimate for 2015 is much lower; about $4 million. While GNI per capita is higher in the U.S. than across the OECD countries, the effect of income alone is likely to be smaller than implied by the large difference between the estimates. The difference is also attributable to the divergent approaches used to select studies and combine the results.

### 3.2 Adjustment for income differences

When applying these estimates in other settings, a key question is the extent to which they should be adjusted for variations in the risks and populations affected. The consensus in the reviews and guidance documents referenced above is that the available evidence is not sufficient to support adjustment for most differences. The one exception is income. Many, if not most, guidance documents and other reviews suggest that these estimates should be adjusted for differences in population-average income across countries and over time.^7,8^

Because these values represent the trade-off between spending on mortality risk reductions and spending on other things, it would be nonsensical to expect that the values would be the same for individuals with substantially different income levels. For example, as illustrated in Table 1, the U.S. VSL estimates suggest the average U.S. resident is willing to pay amounts approaching $1000 dollars for a 1 in 10,000 mortality risk change, equivalent to about 1.6 to 1.7 percent of GNI per capita. In lower-income countries, this sum would represent much or all of an individual’s yearly income.^9^ It seems implausible or impossible that an average individual in these countries would be willing to spend such a large sum on such a small risk reduction, given other essential needs. Overall, individual WTP per unit of risk reduction is expected to decrease as income decreases, resulting in a smaller VSL.

To extrapolate values across countries, analysts select an estimate (or estimates) of the degree of change in the VSL associated with a change in income; i.e., the VSL income elasticity. Although comparisons among high-income populations often find that changes in the VSL are less than proportional to changes in income (an income elasticity of less than one), comparisons between populations with large income differences often find that changes in the VSL are more than proportional to changes in income (an income elasticity of greater than one). An income elasticity greater than one implies that the ratio of VSL to GNI per capita is smaller in lower-than in higher-income populations. This seems reasonable given that lower-income individuals must devote a larger share of their incomes to necessary or urgent expenses.

Adjusting a base VSL for income differences requires an income estimate for the population to which the base VSL applies, an income estimate for the target population, and an estimate of the rate at which VSL changes as income changes; i.e., the average elasticity over the relevant income range. The formula is:

$$VSL_{t\arg et}=VSL_{base*}{\left(Income_{t\arg et}/Income_{base}\right)}^{elasticity}.$$ (1)

It is often convenient to work with ratios of VSL to income rather than VSL itself. This is in part because working with ratios avoids the need to convert values to a common year.^10^ Some also find ratios easier to understand and apply. Derived from the equation above, the relationship of the ratios is:

$$\left(VSL_{t\arg et}/Income_{t\arg et}\right)={(VSL_{base}/Income_{base})*\left(Income_{t\arg et}/Income_{base}\right)}^{\left(elasticity-1\right)}.$$ (2)

The same formulas can be used to extrapolate these values over time within the same country. In that case, the base VSL and income level are for the starting year, and the target VSL and income level are for a future year. Commonly, analysts assume that the same elasticity estimates apply over time as across different populations at the same point in time.^11^

Changes in the income elasticity can change the results by orders of magnitude. Hammitt and Robinson (2011) report that the then-existing studies found VSL income elasticities ranging from as low as 0.1 to greater than 2.0. More recent reviews seem to be coalescing around estimates closer to 1.0 (OECD, 2016; World Bank and IHME, 2016; Viscusi & Masterman, 2017*a*,*b*; Masterman & Viscusi, 2018), generally recommending elasticities around 0.8 for extrapolating across high-income countries and between 1.0 and 1.2 for lower-income countries.

There is, however, a substantial difference in the base VSLs recommended in recent work, which leads to large differences in the country-specific VSLs that result. The OECD and the World Bank rely on estimates from the OECD (2012) meta-analysis of stated-preference research introduced earlier, supplemented by additional studies. The series of studies co-authored by Viscusi and Masterman instead rely on the results of a meta-analysis of U.S. wage-risk estimates that yields a much higher VSL, similar to the estimates currently used by U.S. regulatory agencies.^12^

Table 2 summarizes the approaches recommended in these studies, which vary in whether they use GDP per capita or GNI per capita, and use exchange rates or purchasing power parity, when transferring values. For high-income countries, there is often relatively little difference between income measured using exchange rates or purchasing power parity. For lower-income countries, the choice between exchange rates and purchasing power parity can substantially affect the estimates, regardless of whether the exchange rate is for a single year or integrates data from multiple years as in the World Bank’s Atlas method. For example, as reported by the World Bank, in 2015 GNI per capita for India (a middle-income country) was $6060 if estimated using purchasing power parity, but $1600 if measured using exchange rates and the Atlas method. For Malawi (a low-income country), GNI per capita was $1120 based on purchasing power parity and $340 using exchange rates and the Atlas method. Because Viscusi and Masterman rely on exchange rates, their VSL estimates will be much smaller for lower-income countries than the values that would result if the same approach was applied to income measured using purchasing power parity.^13^ We return to this range of values in developing our recommendations for sensitivity analysis in Section 6.

**Table 2:** Recent recommendations for estimating VSL in low- and middle-income countries.^a^

Report	Central base estimates^b^			Central income elasticity estimates^b^	
	Source	VSL (dollar year)	Income level (measure)	Source	Elasticity
OECD (OECD, 2016)^c^	OECD (OECD, 2012)	$3.0 million (2005 USD)	Not reported (OECD GDP per capita, PPP)	OECD (2012) meta-analysis and additional review	Low income: 1.0 Middle income: 0.9 High income: 0.8
World Bank and IHME (2016)^c,d^	OECD (2012)	$3.83 million (2011 USD)	$37,000 (OECD GDP per capita, PPP)	Narain and Sall (2016) review	Low and middle income: 1.2 High income: 0.8
Viscusi and Masterman (2017b)	Viscusi (2015b)	$9.6 million (2015 USD)	$55,980 (U.S. GNI per capita, MER)	Viscusi and Masterman (2017b) meta-analysis	All income levels: 1.0
Masterman and Viscusi (2018)	Viscusi (2015b)	$9.6 million (2015 USD)	$55,980 (U.S. GNI per capita, MER)	Masterman and Viscusi (2018) meta-analysis	GNI per capita < $8809: 1.0 GNI per capita F020≥ $8809: 0.85^e^

Notes: PPP = purchasing power parity, MER = market-exchange rate.

a The World Bank income categories are based on GNI per capita measured in U.S. dollars using the Atlas method, which smooths exchange rate fluctuations by relying on a three-year moving average, price-adjusted conversion factor. As of 2017, the categories were defined as follows based on 2015 GNI per capita: low income, $1025 or less; lower middle income, from $1026 through $4035; upper middle income, from $4036 through $12,475; high income, $12,476 or more.

b Central or recommended best estimates highlighted in each study; each also examines uncertainty in the elasticity estimates.

c Income groups defined using World Bank categories.

d The Lancet Commission on Pollution and Health (Landrigan et al., 2018) uses the same approach in estimating the economic burden of pollution globally.

e From base model recommended for use in inter-country transfers by the authors.

These recommendations reflect a substantial division in the literature. In the OECD and World Bank reports, a significant concern is the reasonableness of the resulting estimates; i.e., whether the estimates that result from combining the base OECD VSL with the elasticities appear consistent with VSL estimates from selected studies conducted in lower-income countries. In the Viscusi and Masterman studies, the primary concern is publication-selection bias.

The Viscusi and Masterman studies rely on global sets of VSL studies and use meta-analysis to combine the results. Viscusi and Masterman (2017*b*) consider wage-risk studies; Masterman and Viscusi (2018) consider stated-preference studies (including those identified in the review discussed later in this paper). The authors reject reliance on base values from studies conducted in lower-income countries due to concerns about publication-selection bias. However, they use the results of the full set of studies to explore income elasticity in meta-analysis that adjusts for such bias. In their analysis of wage-risk studies, Viscusi and Masterman recommend using an elasticity of 1.0 because “it is tractable and because we fail to reject the hypothesis that the international elasticity is equal to 1.0 in any of our specifications” (Viscusi & Masterman, 2017*b*, p. 244). In their study that relies on stated-preference research, Masterman and Viscusi (2018) suggest instead using a two-step function, based on their analysis of elasticities across income groups, as illustrated in Table 2.

However, all of these recommendations face the same limitation. There is very little research from low- and middle-income countries available that can be used to develop and validate these approaches. Few, if any, of the studies considered by the research teams were conducted in low-income countries; the studies conducted in middle-income countries address only a small subset of these nations.^14^ We explore these studies below.

## 4 Review of research conducted in low- and middle-income countries^15^

To supplement the above analyses, we reviewed studies conducted in low- and middle-income countries in more detail. Our starting point was the research identified in previous reviews, including those discussed earlier. We then searched the literature for studies conducted in the 172 countries categorized as low- or middle-income by the World Bank in any of the past 20 years (1997 through 2017). We also contacted researchers to identify additional work. We first describe our selection criteria and the resulting studies, then discuss the relationship between the estimated VSL and income.

### 4.1 Selection criteria

**Figure 1:**
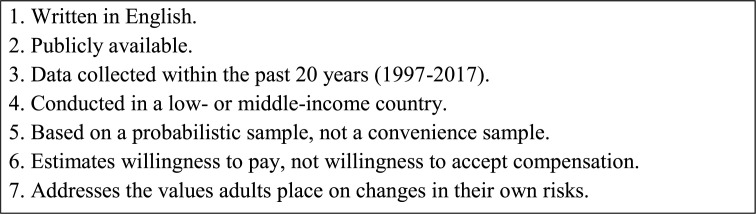
Selection criteria.

To select studies for detailed review, we rely on a series of criteria designed to ensure that the studies are of reasonable quality and suitable for use when considering how to best transfer estimates across countries, listed in Figure 1. We build upon the criteria used in previous reviews, but adapt the criteria to reflect our focus on low- and middle-income countries.^16^

Criteria 1 and 2 (written in English, publicly available) align with the goals of this work: to develop methodological recommendations for application in policy analysis. To achieve this goal, the underlying studies should be accessible to those conducting and reviewing the analyses. While English is not necessarily the first language of those involved, it is often used in academic discourse and publications and is the language most likely to be understood by a wide range of researchers. To ensure that stakeholders and others interested in the analytic results can access the underlying research, we consider only publicly-available sources including peer-reviewed journal articles, working paper series maintained by academic and other institutions, and reports from government agencies and international organizations.

Criteria 3, 4, and 5 (data collected within the past 20 years in low- or middle-income countries using a probabilistic sample) reflect our interest in understanding the preferences of these populations. Older studies are less likely to reflect the preferences of those affected by current or future policies, and also do not reflect researchers’ evolving understanding of how to best conduct these studies. We consider studies that rely on probability samples due to our desire for values that are representative of the population studied.

Criteria 6 and 7 relate to the need for values that measure a reasonably consistent outcome for comparability. Criterion 6 is primarily relevant to stated-preference studies, and requires that they elicit WTP rather than WTA.^17^ WTP is more often used in benefit-cost analyses because policy options typically involve expenditures for improvements from the status quo rather than compensation for damages. WTP is also more frequently studied and the estimates are generally considered more reliable; the reasons for the large and variable differences between estimated WTP and WTA are not well understood (Horowitz & McConnell, 2002; Tunçel & Hammitt, 2014). Criterion 7 focuses on changes in an adult’s own risk consistent with the conceptual framework for benefit-cost analyses, which assumes that the individual is the best or most legitimate judge of his or her own welfare.^18,19^

We found 17 stated-preference studies (which include 18 surveys) and nine wage-risk studies that meet our selection criteria. These studies vary in the types of mortality risks they consider, including transportation, environmental, and occupational risks as well as risks from unspecified causes. These 26 studies were conducted in 15 countries, all of which are now middle- or high-income. Hence the available studies represent the preferences of only a small fraction of the population globally. The studies and countries addressed are listed in Appendix A.

### 4.2 Study quality

Evaluating the quality of these 26 selected studies is difficult, in part because they vary in the extent to which they document the data sources and methods used and in part because there are few studies that address similar populations – which makes it challenging to determine whether differences in results are due to differences in the populations or other study characteristics.

For stated-preference studies, one indicator of validity is whether estimates of WTP are sensitive to scope; i.e., whether WTP for different magnitudes of risk reduction varies by a statistically significantly amount. This sensitivity is of particular importance for both conceptual and practical reasons. Theory suggests that WTP should be larger for a larger risk reduction, and close to proportional to the risk change as long as WTP is small relative to income (see Corso et al., 2001; Alolayan et al., 2017). The common practice of applying a constant VSL across differently-sized risk changes rests on this assumption of proportionality; if WTP is not proportional to the risk change, then estimated VSL depends on the magnitude of the risk change.^20,21^

Using the same WTP for differently-sized risk reductions would suggest that investing in policies that provide smaller risk reductions may be preferable (assuming the costs of implementing the policy increase with the size of the risk reduction), which seems nonsensical. It is more likely that individuals are misinterpreting the probabilities.^22^ This misunderstanding can be reduced by including educational materials and tests for probability comprehension in the survey.

The 17 stated-preference studies we identify include 18 surveys, of which ten test whether WTP differs across risk reductions of different magnitudes. Six find that WTP is significantly different, but it is often less than proportional to the risk change. The lack of scope tests in many of these studies is troubling, suggesting that researchers may not fully understand some of the challenges associated with conducting stated-preference research. These tests help validate whether respondents comprehend the outcome to be valued and can be seen more generally as an indicator of whether the researchers adhere to standards for high-quality work.

For the wage-risk studies, work conducted in the U.S. suggests that the results of these studies may be very sensitive to the quality of the risk data used and to the controls included in the statistical models (see, for example Viscusi, 2013). Inspection of the information reported in the articles suggests that several studies from lower-income countries may have significant drawbacks. This finding is perhaps not surprising given that relatively sophisticated and resource-intensive data collection systems are required to provide high-quality data on the parameter values. For example, substantial confirmation is often needed to ensure that deaths categorized as work-related are in fact associated with the job rather than resulting from other causes. In addition, ideally the analysis would use risk data categorized by both occupation and industry, since industry-level data aggregates across individuals facing significantly different risks (e.g., including both administrative assistants and forklift operators in the construction industry category). Data are also needed to control for the impact of non-fatal risks on wages as well as other influencing factors.

Because job-related deaths are relatively infrequent, it is usually desirable to rely on risk data from more than one year. These studies also require combining data from different sources; e.g., one that reports job-related deaths, and another that reports worker characteristics (such as income) by industry or occupation. If these sources address different populations (e.g., one is national and another is regional), the results may be biased. Of the nine wage-risk studies, two only consider data collected in a single year, and two do not provide sufficient information to explore the match between the data sources used. While the remaining articles suggest that the match may be reasonable, more investigation of the underlying data sources would be needed to confirm this conclusion as well as to determine their overall quality.

### 4.3 Relationship to income

Typically, VSL estimates are transferred across countries based on estimates of population-average income. In this section, we discuss the relationship of the values in the studies identified above to income levels in the countries where they were conducted. For illustrative purposes, we rely on one estimate from each study; either the best estimate highlighted by the authors or the midpoint of the estimates if more than one is highlighted. While we recognize that this approach means that our results reflect the biases of the authors (as discussed in Viscusi, 2018), such simplicity seems warranted given the other issues associated with these calculations as described below. Masterman and Viscusi (2018) include all estimates that the authors of each study choose to report in their meta-analysis described in Section 3.

As noted earlier, our work focuses on estimating population-average values for individual low- and middle-income countries, using estimates of GNI per capita (adjusted for purchasing power parity). However, GNI (as well as GDP) per capita is likely to differ significantly from the income of the individuals studied for two reasons. First, many studies are not based on national samples and the mean income of those studied may be substantially different than the national average. Second, the data collected by most researchers reflects individual or household income, which is a different measure than GNI or GDP per capita (see Hammitt, 2017, for more discussion).

Review of the data reported in the 26 selected studies suggests that the mean income level of the respondents differs significantly from estimates of GNI per capita (see Appendix B). Of the 26 studies, 21 report mean income levels for the sample. Of these 21, seven report household income, eight report individual income, two report both household and individual income, and four do not indicate whether the income level is for the individual or the household. For the 10 studies where mean individual income is reported, in six studies it is within ±50 percent of GNI per capita for the country. In the remainder, mean individual income for the sample varies from about 29 percent of GNI per capita to about 290 percent. These data raise serious questions about using these estimates to develop population-average values for each country. Thus the common practice of using GNI (or GDP) per capita to estimate income when transferring values across countries may introduce substantial error.

We ignore this problem for the moment and compare the ratio of VSL to GNI per capita for a subset of the 26 studies. We focus our comparison on a subset because several studies report VSLs that result in implausibly high or low ratios, as reported in Appendix B. It seems highly unlikely that the ratio in substantially poorer countries would exceed the ratio for the U.S., which is about 160 as illustrated in Table 1. Exactly where we set this upper bound makes little difference, since we would exclude the same studies using any threshold between about 140 and more than 300. We also exclude those where the ratio is less than 20.^23^ This lower bound reflects the expectation that the VSL would exceed the future earnings (and consumption) of the average individual; we assume that an adult of average age would have at least 20 years of life remaining in the countries of concern. Note that these ratios should not be viewed as an indicator of the quality of these studies; as discussed above, GNI per capita may differ significantly from the income levels of those included in each study.

Our starting point is the 26 studies discussed in the previous sections. The results from seven of the 18 stated-preference surveys are outside of these upper and lower bounds, as are the results from five of the nine wage-risk studies. Thus in the analysis that follows, we exclude the results of these 12 studies, focusing on the 15 remaining estimates.

**Figure 2:**
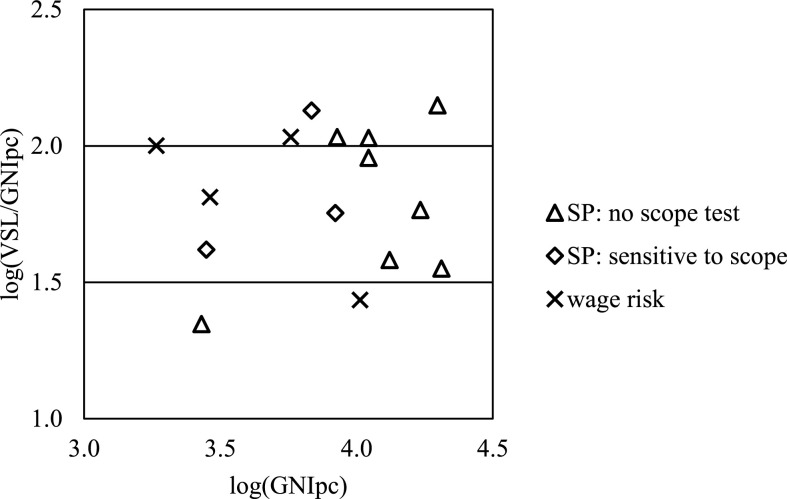
Ratio of VSL to GNI per capita (GNIpc).^24^

Figure 2 displays the ratios of estimated VSL to GNI per capita (in the same year as the VSL estimate) for the remaining 11 stated-preference surveys and four wage-risk studies. The scales are logarithmic (base 10). Overall, there is substantial variability in the ratio of VSL to GNI per capita, with most falling between roughly 40 (log ratio = 1.6) and 107 (log ratio = 2.0). The ratios appear similar for the four wage-risk studies (log ratios = 1.4, 1.8, or 2.0) and the 11 stated-preference studies (log ratios = 1.4 to 2.2), regardless of whether the latter test for sensitivity to scope. Of the studies in our review that fell within the plausible range of VSL to GNI per capita ratios (and hence are included in the figure), all that conducted a scope test found that WTP was sensitive to scope.

An alternative way to view the implications of these results is to estimate the income elasticity that would be needed to extrapolate from a VSL estimate for a high-income country to each of these VSL estimates. We rely on the U.S. Department of Health and Human Services (USDHHS) estimate in our comparisons because it is derived from a recent review and includes evaluation of both stated- and revealed-preference studies. We update the estimate to 2015 dollars, following the approach described in U.S. Department of Health and Human Services (2016).^25^ This yields a central VSL estimate of $9.4 million, or 162 times U.S. GNI per capita ($57,900) in that year. Relying instead on the USEPA or USDOT values, or on the values recommended in the Viscusi and Masterman studies, would lead to very similar results.

Again excluding implausibly high and low ratios, the income elasticity implied by comparing each of the remaining 15 ratios (reported in Appendix B) with the ratio for the USDHHS estimate ranges from 1.1 to 2.6 with a mean of 1.5 and a median of 1.4.^26^

These results seem reasonable, given that we expect that WTP for small changes in mortality risk will decline as income decreases. However, this comparison highlights the problems associated with using this literature as the basis for extrapolating VSL estimates across countries. First, as discussed in the previous sections, these studies were conducted in relatively few countries (primarily middle-income) and have several important limitations. Second, the mean income levels of the samples are not consistently reported, and may vary substantially from the estimates of GNI per capita (as well as GDP per capita) often used in cross-country transfers. Third, there is no clear relationship between VSL and income across these studies. Finally, regardless of whether the income measure used in these transfers is the sample mean reported by the authors or GNI or GDP per capita, the reliance on income to estimate inter-country variation ignores the cultural and other factors that may significantly influence these values.

It is difficult, if not impossible, to improve these estimates or validate these results without more high-quality research from low- and middle-income countries, which can be compared to the results of extrapolating from an estimate for a high-income country using alternative elasticities. More work is also needed to better understand how factors other than income affect these values.

It is unclear whether the uncertainty in the resulting VSL estimates is large relative to the uncertainty in other parameter values used in benefit-cost analysis. In some cases, the estimates of net benefits may be sensitive to these values; in others, whether a policy yields net benefits or which policy yields the greatest net benefits may not change regardless of which value is used.^27^

## 5 Adjustments for age and life expectancy

The estimates featured above are population-average values for adults, although the age range included in each study differs somewhat. Because the number of life years remaining for younger or older individuals may be much larger or smaller respectively, intuition suggests that different values may be applicable. However, both theory and empirical work indicate that the relationship is uncertain (e.g., Hammitt, 2007). Research conducted largely in high-income countries suggests that values for children may exceed the average for adults by perhaps a factor of two (e.g., Robinson, Raich, & Hammitt, 2018), values for working age adults may follow an inverse “U” pattern that peaks in middle age (e.g., Aldy & Viscusi, 2007), and values at older ages may remain constant or decrease (e.g., Krupnick, 2007). However, the results across studies are inconsistent and raise questions about the robustness of these findings. For low- and middle-income countries, little empirical research is available and it is unclear whether the same patterns hold.

In applied work, one frequently used simplifying assumption is that the value of mortality risk reduction increases with life expectancy (or decreases with age). To implement this approach, often a constant VSLY is used, derived from a VSL using simple assumptions. Typically, VSL is divided by the (discounted) life expectancy of an individual at the average age of those studied (see Jones-Lee et al., 2015, for more discussion). This VSLY is then multiplied by the change in (discounted) life expectancy associated with the policy to estimate the value of mortality risk reductions for individuals in different age groups. For individuals of average age, the results will be the same as the results of applying the VSL; the results will be greater for younger individuals and smaller for those who are older. Assuming VSLY is constant provides a rough proxy for the effects of age and life expectancy, but is not well-supported by theory or the available empirical research.

One question that arises in this context is whether future life years should be discounted at the same rate as money values. The logic for discounting monetary values is that one can shift money through time via saving or borrowing at a positive interest rate. But life years cannot necessarily be shifted through time, so there is no parallel argument for discounting; the appropriate discounting depends on individual preferences for years lived or health experienced at different times.

Some argue that individuals should not discount their own future well-being; doing so is often categorized as a failure of self-control (e.g., not exercising or dieting now, compromising future health and longevity). Empirical evidence about how individuals value years of life at different ages is provided by Nielsen et al. (2010) and Hammitt and Tunçel (2015). Both studies consider mortality risk reductions that differ in timing or duration; e.g., whether the risk reduction is one-time or ongoing. They find significant heterogeneity in preferences, indicating that whether individuals discount future life years at a positive, negative, or zero rate varies. These findings suggest that future life years should not necessarily be discounted at the same rates as monetary values.

Benefit-cost analyses conducted in low- and middle-income countries must at times also address deaths around the time of birth, which raises difficult normative questions as well as empirical issues. We know very little about parental WTP to avert the death of a fetus or a newborn. One option is to apply the VSL and VSLY estimates described above to deaths that occur at or shortly after birth (applying the VSLY estimate to life expectancy at age zero), and to value deaths that occur prior to birth at zero. Additional sensitivity analysis is likely to be desirable that tests the effects of assigning positive values to deaths prior to birth.

## 6 Recommendations and priorities for future research

Ideally, the value of mortality risk reductions in low- and middle-income countries would be derived from multiple high-quality studies of the population affected by the policy, given the likelihood that these values will vary depending on characteristics of the society, the individuals affected, and the risk. However, we expect extrapolation from studies of other populations will continue to be necessary in the near term, given the paucity of studies conducted in these countries.

Below, we describe near-term recommendations for developing population-average estimates and for assessing the effects of differences in age or life expectancy. We conclude by summarizing longer-term research needs. Analysts addressing policies to be implemented in high-income countries will often have sufficient studies of reasonable quality to develop estimates appropriate for that context; the recommendations that follow are intended for application solely in low- and middle-income countries.

Our near-term recommendations focus on the effects of income and life expectancy and do not address other differences between the risks and populations studied and the risks and populations addressed by the analysis. These other differences should also be explored both qualitatively and quantitatively. Analysts should highlight the implications for decision making; i.e., the extent to which the uncertainties affect whether a policy has positive net benefits or the ranking of alternative policies.

### 6.1 Population-average values

In the near term, to ease comparison with the findings of other benefit-cost analyses as well as examine related uncertainties, our recommendations for estimating the VSL in low- and middle-income countries include selecting a preferred estimate and conducting a standardized sensitivity analysis using common defaults.

The value featured as the preferred estimate should reflect the decision-making context, taking into account the characteristics of the individuals affected by the policy (such as income and age) and of the risk that the policy addresses (such as whether it results from illness or injury or is viewed as voluntarily incurred or under the individual’s control). Ideally, these values should be derived from a criteria-driven review of the WTP literature which identifies high-quality studies that are suitable for the context, including the characteristics of the risks and of the affected population. Meta-analysis and other methods are often helpful in synthesizing the results across studies.

The selection criteria and studies discussed in this paper provide a starting point for such a review. In addition to searching for more recent studies, analysts should review other sources for more information on best practices when developing criteria. For example, Johnston et al. (2017) discuss best practices for stated-preference studies and Viscusi (2013) identifies the issues that may arise in conducting wage-risk studies. Neither source focuses specifically on estimating VSL in low- or middle-income countries; analysts will need to exercise judgment in applying the recommendations to the settings of concern.

Regardless of whether context-specific values are available, analysts should conduct a standardized sensitivity analysis to facilitate comparison to other studies and to explore the effects of uncertainties. The sensitivity analysis should follow the current practice of extrapolating a country-level population-average VSL estimate from the substantial research conducted in high-income countries, using data on GNI per capita (measured in international dollars using purchasing power parity) to estimate income, and an assumed income elasticity. The results should be reported in the local currency as well as in international dollars, to facilitate comparison to costs and to other policies that could be or have been implemented in that country.

The sensitivity analysis should use the following three estimates; option (a) is generally the preferred default, while options (b) and (c) are designed to align the results with the range applied in other research (see Table 2) and explore related uncertainties.

(a) VSL extrapolated from a U.S. estimate to the target country using an income elasticity of 1.5. The starting point for this calculation should be a U.S. VSL to GNI per capita ratio of 160, based on a U.S. VSL of $9.4 million and U.S. GNI per capita of $57,900. If this approach yields a value for the target country less than 20 times GNI per capita for that country, then 20 times GNI per capita should be used instead.
(b) VSL = 160 * GNI per capita in the target country. This calculation applies the U.S. ratio to all countries, which is equivalent to using that ratio as the starting point and assuming income elasticity is 1.0.
(c) VSL = 100 * GNI per capita in the target country. This calculation applies the OECD ratio to all countries, which is equivalent to using that ratio as the starting point and assuming income elasticity is 1.0.

Option (a) addresses concerns about the resources available for spending on mortality risk reductions in low- and middle-income countries. It seems reasonable to expect that the proportion of income devoted to attaining these small risk reductions will decrease as income decreases, rather than remain constant. More research on the values held by low- and middle-income populations is needed to estimate the rate of decrease with greater precision. In the interim, we recommend relying on the mean elasticity estimate (1.5) found when extrapolating from a U.S. value to the values found in our review in Section 4.3. While this extrapolation is subject to several limitations, an elasticity of 1.5 seems reasonable given the substantial income differences. The resulting VSLs should be limited to a lower bound of 20 times GNI per capita, to reflect the expectation that the population-average VSL will not be less than expected future income over the years of life remaining for the average adult.

Options (b) and (c) are designed to facilitate comparisons to studies that rely on approaches recommended in previous work as well as reflect uncertainties in the available research. The use of a constant VSL to GNI per capita ratio under both options is equivalent to assuming that income elasticity is 1.0 when extrapolating from a U.S. or OECD base value to a low- or middle-income country.

The first multiplier (160) is derived from the recommended U.S. values ($9.4 million VSL, $57,900 GNI per capita), rounded to two significant figures. It is similar to the ratio of the U.S. values used by other regulatory agencies and by Viscusi and Masterman in their exploration of publication-selection bias. Combining this base value with an elasticity of 1.0 likely provides a high-end estimate of the values for low- and middle-income countries.

The second multiplier (100) is derived from the OECD meta-analysis of stated-preference studies conducted globally. The studies that use this value as a starting point (OECD, 2016 and World Bank and IHME, 2016) rely on elasticities ranging from 0.8 to 1.2 depending on the study and the country; combining a base ratio of 100 with an elasticity of 1.0 essentially splits the difference. While the results are always lower than the values estimated under option (b), the relationship to the values estimating using option (a) depend on the income level of the target country.

We illustrate the results in Table 3, which summarizes the ratio of VSL to GNI per capita using these alternative approaches. In this example, we use six income levels that span the range of income levels found in low- and middle-income countries when expressed as GNI per capita based on purchasing power parity. As expected, for low-income countries, the estimates using an elasticity of 1.5 are much smaller than the estimates using the other approaches; for middle-income countries, the range is narrower. Estimates using each of these three approaches for all countries categorized as low- or middle-income (based on 2015 GNI per capita) are provided in a supplement posted on the journal’s website.

**Table 3:** Examples of extrapolated VSL estimates using alternative approaches.

Approach	**GNI per capita (2015 international dollars)**					
	$1000	$5000	$10,000	$15,000	$20,000	$25,000
Base ratio = 160 Elasticity = 1.5	$0.02 million (21 * GNI pc)	$0.24 million (48 * GNI pc)	$0.67 million (67 * GNI pc)	$1.2 million (83 * GNI pc)	$1.9 million (95 * GNI pc)	$2.7 million (110* GNI pc)
Base ratio = 100 Elasticity = 1.0	$0.10 million (100* GNI pc)	$0.50 million (100* GNI pc)	$1.0 million (100 * GNI pc)	$1.5 million (100 * GNI pc)	$2.0 million (100 * GNI pc)	$2.5 million (100 * GNI pc)
Base ratio = 160 Elasticity = 1.0	$0.16 million (160* GNI pc)	$0.80 million (160* GNI pc)	$1.6 million (160 * GNI pc)	$2.4 million (160 * GNI pc)	$3.2 million (160 * GNI pc)	$4.0 million (160 * GNI pc)

Note: All results rounded to two significant digits.

It often requires several years for policy impacts to fully manifest. Analysts should also project the change in real income (measured as GNI per capita) in the country of concern that occurs over this time period and adjust the VSL estimates accordingly, using the approaches above. The underlying assumption is that income elasticities are the same over time as across different income groups at the same point in time.

These recommendations should be periodically revisited and revised to reflect the results of new research.

### 6.2 Adjustments for age and life expectancy

The approaches discussed above yield population-average estimates for an average-age adult. If the policy disproportionately affects the very young or the very old, analysts should also conduct sensitivity analyses using VSLY estimates. In such cases, analysts should derive a constant VSLY from each of the VSL estimates discussed above; i.e., the context-specific estimates (if any) and the three estimates that result from the standardized sensitivity analysis. Note that if the mean age of the individuals affected is the same as the mean age used in deriving VSLY from VSL, the results of applying each approach should be identical and this sensitivity analysis is not needed.

This constant VSLY should be calculated by first estimating the population-average VSL for the country affected by the policy, then dividing that VSL by undiscounted future life expectancy at the average age of the adult population in that country. In this calculation, “adults” would ideally be defined by the age range during which individuals are most likely to participate in the labor force, for consistency with the age ranges often included in the underlying VSL research. However, due to the difficulties inherent in defining this average age in some countries and the desire to promote consistency, analysts may wish to rely on the age that is equivalent to one-half of life expectancy at birth as a rough proxy. The constant VSLY that results should then be multiplied by the change in future life expectancy for those affected by the policy.

This calculation should not discount future years for two reasons. First, individuals may discount their own future years at a rate smaller than the rate at which they discount future consumption or other monetary values. Second, calculating VSLY using discounted future life years flattens the relationship between the value of reducing risk and age, making it more similar to the alternative of using the same VSL for all ages. For sensitivity analysis, it seems preferable to maintain the full effect of valuing life years equally rather than moderating the effect through the choice of some positive discount rate.

For example, if the population-average VSL for the country is $0.9 million and the life expectancy of an adult of average age in that country is 30 years, then the VSLY would be $30,000 based on simple division. Ideally, analysts would instead use a life table in this calculation, that indicates the likelihood of surviving each year of age conditional upon reaching that age. If the policy extends the life expectancy of individuals in the affected population by 10 years, the total value per individual affected would be $300,000 (10*$30,000).

If the analysis addresses deaths around the age of birth, the VSL and VSLY estimates described under the above recommendations can be used. However, analysts should also explore the impact of assigning positive values to mortality risk reductions that occur prior to birth.

### 6.3 Long-term recommendations

Over the longer term, more research is needed that explicitly addresses the value of mortality risk reductions in low- and middle-income countries. To support and encourage such studies, research methods tailored to this context should be further developed.

1. *Conduct additional research on WTP for mortality risk reductions in low- and middle-income countries:* Substantial additional research is needed on the value of mortality risk reductions in these countries, given the importance of these estimates in policy analysis and the likely differences in preferences across members of different populations.
2. *Develop protocols for the conduct of these studies that are tailored to low- and middle-income settings*. To encourage additional research and ease its implementation, more work is needed on developing approaches for data collection and analysis that can be feasibly implemented in low- and middle-income settings, which are designed to provide reasonably valid and reliable results. Such approaches should be tailored to the resources available for this type of research and should take into account the characteristics of these populations as well as the risks they face.
3. *Develop an easily accessible repository for valuation studies*, that includes primary research as well as research that synthesizes the results. Many researchers in low- and middle-income countries may not have easy access to these studies, so will otherwise find it difficult to conduct the careful review that is needed to determine whether studies are applicable in particular settings and to form the foundation for new research.

Such additional research will help analysts, decision makers, and other stakeholders better understand the preferences of those affected, which can aid in policy implementation as well as evaluation. It also moves away from focusing largely on the effects of income differences, and encourages greater attention to other sources of variation such as differences in cultural norms and other context-specific factors.

## Footnotes

1 In the United Kingdom, VSL is usually described as the value of a prevented fatality (VPF), and the value per statistical life year (VSLY) as the value of a life year (VOLY). We use the term value “per” statistical life rather than value “of” statistical life to emphasize that these estimates reflect a rate of exchange.

2 The approaches discussed in this paper are designed largely for application in a microeconomic (partial equilibrium) framework; i.e., to assess policies that are unlikely to substantially affect market prices or the economy at large. Jamison et al. (2013) instead address macroeconomic effects, applying a “full income” approach which adds the value of mortality risk reductions to the value of changes in gross domestic product (GDP). Our recommendations are similar to those used in a recent study of the economic burden of pollution (Landrigan et al., 2018), but that study considers much larger impacts.

3 Estimates of willingness to accept compensation (WTA) are also consistent with the benefit-cost analysis framework. However, WTP is estimated more frequently in empirical research and typically used to value improvements from the status quo. See Viscusi (2015*a*), Hammitt (2015), and Knetsch (2015) for more discussion.

4 Unless otherwise noted, all GNI per capita estimates are expressed in current international dollars based on purchasing power parity as reported by the World Bank (https://data.worldbank.org/indicator/NY.GNP.PCAP.PP.CD?locations=US).

5 Email from Ståle Navrud, February 8, 2018.

6 In addition to being a census rather than a sample, the CFOI involves substantial efforts to confirm the data.

7 In this paper, we focus on national estimates. As discussed in our companion paper on distributional analysis (Robinson, Hammitt, & Adler, 2018), these estimates should be adjusted for within-country income differences in cases where the risk reductions accrue to subpopulations with income levels that differ from the national average.

8 Some have argued that national, population-average estimates should be used regardless of the income of those affected, perceiving the use of varying estimates as inequitable. However, using the same value for different income groups ignores the variation in the values that each group may place on the risk reductions it receives, potentially leading to inaccurate conclusions regarding their preferences as well as regarding the net benefits of the policy.

9 As of 2017, the World Bank defined low-income economies as those with 2015 GNI per capita of $1025 or less; lower middle-income economies as those with GNI per capita between $1026 and $4035; upper middle-income economies as those with GNI per capita between $4036 and $12,475; high-income economies as those with GNI per capita of $12,476 or more. GNI per capita in this case is measured in U.S. dollars based on market-exchange rates, using the Atlas method. Using the same method, U.S. 2015 GNI per capita was estimated as $56,300. This value differs from the U.S. estimate presented in the text because the Atlas method uses a three-year moving average, price-adjusted conversion factor to smooth exchange rate fluctuations.

10 Generally, values should be inflated in the original currency to the analytic base year, using an inflation rate that reflects conditions in the country where the study was conducted, prior to converting to other currencies.

11 As noted earlier, we focus on population-average values for low- and middle-income countries. The same equations can be used to adjust the estimates for differences in income levels within a country, which may be of particular interest when a policy affects individuals with income levels that differ significantly from the national average.

12 The income elasticity implied by the difference between the US and OECD VSL in Table 2 is 2.2.

13 If income elasticity is calculated from VSL and income estimates measured using purchasing power parity, the elasticity will generally be further from 1.0 than when VSL and income are measured using market exchange rates, coinciding only if the elasticity is 1.0.

14 Of the 68 wage-risk studies in Viscusi and Masterman (2017*b*), only two were from middle-income countries, based on the World Bank’s 2015 classifications. The remainder were from countries categorized as high income. Masterman and Viscusi (2018) includes 85 stated-preference studies, including those identified in the review discussed below. The authors note that about 62 percent of the 1145 estimates from these studies that they use in their analysis are from high-income countries and the remainder are from middle-income countries.

15 Our working paper provides more detailed information on this review (Robinson, Hammitt, and O’Keeffe 2018); in this paper we add a study identified by one of the anonymous reviewers (Qin et al., 2013).

16 Our starting point was criteria used in a Robinson and Hammitt (2016) review of U.S. studies, which in turn were based on criteria suggested by independent experts advising the USEPA and USDOT on developing their VSL estimates (U.S. Environmental Protection Agency, 2010*b*; Kling et al., 2011; U.S. Department of Transportation, 2016) as well as other sources.

17 Revealed-preference studies typically address a market equilibrium rather than a change that can be characterized as WTP or WTA. However, Kniesner et al. (2014) find that there is not a significant divergence between WTP and WTA when estimated using revealed preferences for job-related risks.

18 For consistency with the VSL definition (see Section 2), we exclude studies that address outcomes which occur with certainty or that address particular programs or interventions without separating the value of mortality risk reductions from the value of other outcomes.

19 Studies that instead address risks to others (or to the community of which one is a part) raise difficult conceptual and practical issues. For discussion of conceptual issues related to the treatment of altruism in benefit-cost analysis, see Jones-Lee (1991) and Bergstrom (2006). The available research results are often counter-intuitive; some find that WTP for a private risk reduction is higher than WTP for a public program that also benefits others (see, for example, Svensson & Johansson, 2010; Lindhjem et al., 2011). This finding suggests that more work is needed to ensure that respondents understand and accept these scenarios.

20 For example, if WTP is $900 fora1in 10,000 risk change and $4500 fora5in 10,000 risk change, then the VSL (WTP divided by risk change) is $9 million in both cases. If the changes are not proportional, then the VSL differs and it is not clear what VSL is appropriate for small risk changes.

21 While some studies report whether WTP is statistically significantly different for risk reductions of different magnitude (e.g., OECD, 2012), a significant difference does not necessarily mean that WTP is close to proportional.

22 An alternative explanation is that respondents engage in a form of mental accounting that limits the share of income they are willing to spend on reducing mortality risks; if this is the case, these studies provide little guidance for the allocation of resources across different policy interventions.

23 The value of living a year seems likely to exceed the monetary value of consumption because life is about much more than consumption of goods and services. This logic is consistent with the view that the human-capital approach to valuing life-saving (based on lost earnings) provides a lower-bound estimate, as discussed in Hammitt and Robinson (2011). Although this argument is appealing, standard economic models do not imply that VSL must exceed the expected value of future consumption (see Hammitt, 2017).

24 In the figure, the term “scope test” is used to differentiate the stated-preference (SP) studies as follows: those that do not test the sensitivity of WTP to the size of the risk reduction (“no scope test”) and those that conduct a test and find that WTP is sensitive to scope (“sensitive to scope”). None of the studies that remain after excluding those with implausible results both conduct a scope test and find that WTP is insensitive to scope. As noted earlier, scope tests generally cannot be conducted for wage-risk studies.

25 U.S. Department of Health and Human Services (2016) uses the U.S. consumer price index to adjust for inflation, measures income based on workers’ earnings (rather than GNI or GDP per capita), and applies an income elasticity of 1.0.

26 The formula used in this extrapolation is elasticity = 1 + *(*ln*(*ratio_target_/ratio_base_*)/(*ln *(*income_target_/income_base_*)))* where “ratio” is the VSL to GNI per capita ratio for that study and country.

27 For example, in Cropper et al. (2019), the authors consider the mortality risk reductions associated with reducing air pollution emissions from electrical power plants in various locations in India. They apply a wide range of VSL estimates, and find that benefits exceed costs in some areas under all scenarios. For other areas, whether installation of pollution controls leads to net benefits depends on the VSL estimate used.

## Appendix A. Adult VSL studies conducted in low- and middle-income countries

In this appendix, we list the 26 VSL studies which meet the selection criteria described in Section 4 of the paper. The studies are listed in alphabetical order by the country within which they were conducted. More information on our review of these studies is provided in Robinson, Hammitt, and O’Keeffe (2018).

**Table A1:** VSL studies conducted in low- and middle-income countries.

Author, date	Country (current World Bank classification)^a^	Year of data collection
**Stated-Preference Studies**		
Mahmud (2009)	Bangladeshm (lower middle)	2003
Ortiz et al. (2009)	Brazil (upper middle)	2003
Guo et al. (2006)	China (upper middle)	2002
Hammitt and Zhou (2006)	China (upper middle)	1999
Hoffmann et al. (2017)	China (upper middle)	2006
Alberini et al. (2006)	Czech Republic (high)	2004
Bhattacharya et al. (2007)	India (lower middle)	2005
Faudzi et al. (2004)	Malaysia (upper middle)	1999
Faudzi et al. (2013)	Malaysia (upper middle)	2006
Ghani and Faudzi (2003)	Malaysia (upper middle)	1999
Hoffmann et al. (2012)	Mongolia (lower middle)	2010
Giergiczny (2010)	Poland (high)	2005
Mofadal et al. (2015)	Sudan (lower middle)	2013
Chaturabong et al. (2011)	Thailand (upper middle)	2011
Gibson et al. (2007)	Thailand (upper middle)	2003
Vassanadumrongdee and Matsuoka (2005)	Thailand (upper middle)	2003
Tekeşin and Ara (2014)	Turkey (upper middle)	2012
**Revealed-Preference Studies**		
Parada-Contzen et al. (2013)	Chile (high)	2006
Qin et al. (2013)	China (upper middle)	2005
Guo and Hammitt (2009)	China (upper middle)	1995
Madheswaran (2007)	India (lower middle)	2001
Hammitt and Ibarrarán (2006)	Mexico (upper middle)	2002
Rafiq and Shah (2010)	Pakistan (lower middle)	2006
Giergiczny (2008)	Poland (high)	2002
Benkhalifa et al. (2013)	Tunisia (lower middle)	2002
Polat (2014)	Turkey (upper middle)	2011

a Indicates status of country based on World Bank 2017 categories (which relies on 2015 GNI per capita using market-exchange rates and the Atlas method). All studies were conducted in countries classified as low- or middle-income at the time the data were collected.

## Appendix B. Relationship of VSL estimates to GNI per capita

In this appendix, we explore the relationship between the VSL estimates highlighted in each study and the reported income level, as well as the relationship to national GNI per capita. The studies are listed in the same order as in Appendix A.

**Table B1:** Relationship of VSL estimates to GNI per capita.

Author, date	VSL estimate^a,b^	GNIpc	VSL/GNIpc	Reported income^c^	VSL/income
**Stated-Preference Studies**					
Mahmud (2009)	$6273	$1550	4.0^†^	$1345*	4.7
Ortiz et al. (2009)	$3,536,000	$9280	381.0^†^	$10,200	346.7
Guo et al. (2006)^c^	$24,000	$3520	6.8^†^	$2888*	8.3
Hammitt and Zhou (2006)	$10,500	$2630	4.0^†^	$5147e	2.0
Hoffmann et al. (2017)	$474,194	$8360	56.7	15,968*	29.7
Alberini et al. (2006)	$2,788,889	$19,820	140.7	$17,188*	162.3
Bhattacharya et al. (2007)	$117,117	$2810	41.7	$8125	14.4
Faudzi et al. (2004)	$1,000,000	$11,060	90.4	NR	—
Faudzi et al. (2013)	$1,000,000	$17,160	58.3	NR	—
Ghani and Faudzi (2003)	$1,181,818	$11,060	106.9	NR	—
Hoffmann et al. (2012)	$921,167	$6830	134.9	$3573	257.8
Giergiczny (2010)	$5,035,556	$11,740	428.9^†^	$12,458**	404.2
Mofadal et al. (2015)^d^	$60,000	$2700	22.2	NR	—
Chaturabong et al. (2011)	$504,032	$13,210	38.2	NR	—
Gibson et al. (2007)	$914,414	$8490	107.7	$2315**	395.1
Vassanadumrongdee and Matsuoka (2005) (air pollution)	$4,007,896	$8490	472.1^†^	$32,712*	122.5
Vassanadumrongdee and Matsuoka (2005) (traffic safety)	$4,572,114	$8490	538.5^†^	$36,437*	125.5
Tekeşin and Ara (2014)	$726,414	$20,480	35.5	$2742	265.0
**Revealed-Preference Studies**					
Parada-Contzen et al. (2013)	$4,625,958	$13,850	334.0^†^	$6957^e^	665.0
Qin et al. (2013)^f^	$188,000	$2900	64.8	$840	223.8
Guo and Hammitt (2009)	$184,366	$1840	100.2	$2049**	90.0
Madheswaran (2007)	$1,480,392	$2070	715.2^†^	$3959^g^	373.9
Hammitt and Ibarrarán (2006)	$280,000	$10,290	27.2	$4200**	66.7
Rafiq and Shah (2010)	$1,728,978	$3900	443.3^†^	$6161	280.7
Benkhalifa et al. (2013)	$617,700	$5740	107.6	$4514	136.8
Giergiczny (2008)	$3,658,333	$11,740	311.6^†^	$11,997	304.9
Polat (2014)	$211,737	$19,490	10.9^†^	$10,996	19.3

Notes: NR = not reported, GNIpc = gross national income per capita.

† Indicates VSL/GNIpc ratio is below 20 or above 160; these studies are excluded from the comparisons in Section 4.3.

a VSL estimates are the “best” or “central” estimates highlighted by the authors or the midpoint of their highlighted range. All estimates are reported in international dollars based on purchasing power parity for the year in which the data were reported by the authors; monetary estimates have not been updated for inflation and reflect different base years (see Appendix A for year of data collection), so are not directly comparable.

b Multiple VSL estimates are reported in some studies. In six cases (Benkhalifa et al., 2013; Faudzi et al., 2013; Hammitt & Zhou, 2006; Madheswaran, 2007; Rafiq & Shah, 2010; Polat, 2014), we use the midpoint of the reported values. Vassanadumrongdee and Matsuoka (2005) conducted two separate CV surveys, one for air pollution and one for traffic safety, and report two VSL estimates as indicated in the table.

c An asterisk (*) indicates that the authors reported average income only at the household level; a double asterisk (**) indicates the authors did not indicate whether the income measure was per household or for the individual. If the authors report income at both the individual and household level, we include individual income in this table.

d The VSL estimate(s) are reported in U.S. dollars; the authors do not report the exchange rate applied in the analysis.

e Parada-Contzen et al. (2013) report that ln(mean monthly wage) = $12.19, which seems implausibly high, suggesting that annual income averages $197,000. We instead use the income level back-calculated by Viscusi and Masterman from the regression results. Email from Clayton Masterman, March 14, 2018.

f Estimates are from Qin et al. (2013) Table 1, reported in 2000 U.S. dollars. The data were originally reported in 2005 RMB, but average income is not reported for that year and the exchange rate used in not known. Email from Lixing Li, October 4, 2018.

g Annual income calculated as a weighted average of the cities studied, based on the number of respondents from each area.
